# Case of a Singular Variety of Hernia

**Published:** 1827-06

**Authors:** B. C. Brodie

**Affiliations:** St. George's Hospital


### MISCELLANEOUS CASES.

### *Case of a singular Variety of Hernia, treated in* St. George's Hospital, *by* B. C. Brodie, Esq. F.R.S.

Anne Magan, a middle-aged woman, about eleven o'clock in the evening of Saturday, the 21st of April, was seized with pain in the abdomen and sickness. After straining violently in the act of vomiting, she discovered an unusual appearance, which led her to believe that she had suffered a miscarriage, and she sent for a female midwife. On the following day she was seen by Mr. Mullins, of New-street, Dorset-square, who discovered a large portion of the small intestine hanging out of the orifice of the anus. The case being so remarkable, Mr. Mullins recommended that the poor woman should be taken to St. George's Hospital, where she was accordingly admitted about six o'clock in the evening of the 22d of April.

At this time not less than two yards of small intestine, with a corresponding portion of the mesentery, were seen protruding through the anus. The whole mass bore marks of a high degree of inflammation, and the intestine was much distended with air and liquid fæces. On examining the rectum with the finger, it was found that there was a transverse slit on the anterior part of it, about two inches above the anus, through which the protrusion of the small intestine had taken place. On attempting to reduce the protruded intestine, at first it readily re-entered the anus, but, when about one-half of it had disappeared, the reduction became difficult, and about one-fourth part of it could not be reduced at all. In fact, no method could be devised by which even a part of it could be made to pass through the slit in the rectum, so as to resume its natural position in the peritoneal cavity. The pressure of the hand caused the small intestine to ascend into the rectum, where it lay only as long as this pressure was continued; and nothing further in the way of reduction could be accomplished.

Under these circumstances, Mr. BRODIE made a longitudinal incision of the linea alba, about two inches in length, below the umbilicus. The incision was continued through the peritoneum into the cavity of the abdomen; and the fingers being introduced at this opening, by gently pulling the small intestine, that portion of it which had protruded through the slit of the rectum was readily drawn back into the abdomen. It having been ascertained, by examining the rectum with the finger, that the reduction was completed, the edges of the wound in the linea alba were brought together by sutures.*

After the operation, the pulse was scarcely perceptible; the extremities were cold; and the patient was sick, throwing up again immediately whatever she swallowed. In about two hours the pulse was somewhat stronger, and the extremities were warmer; but the restoration of the vital powers was imperfect, and after some hours they again began to fail; and the poor woman died about six o'clock in the evening of Monday, the 23d of April.

On examining the body, the peritoneum generally was found much inflamed, and in many parts covered with a layer of coagulated lymph. That portion of the intestine and mesentery which had formed the protrusion was, however, less inflamed than it had appeared to be previously to the operation. There was a transverse opening in the anterior part of the rectum, without any marks of ulceration in the neighbourhood; whence it was concluded that the opening was the result of accidental laceration.

* During the operation, a part of the small intestine protruded through the wound of the linea alba, but it was readily replaced after the reduction of that which had protruded through the opening in the rectum was completed.

